# Formulation Improvements in the Applications of Surfactant–Oil–Water Systems Using the HLD_N_ Approach with Extended Surfactant Structure

**DOI:** 10.3390/molecules26123771

**Published:** 2021-06-21

**Authors:** Ana M. Forgiarini, Ronald Marquez, Jean-Louis Salager

**Affiliations:** Laboratorio FIRP, Universidad de Los Andes, Mérida 5101, Venezuela

**Keywords:** formulation, normalized hydrophilic–lipophilic deviation HLD_N_, extended surfactant, solubilization, enhanced oil recovery, interfacial tension, drilling fluid, dehydration

## Abstract

Soap applications for cleaning and personal care have been used for more than 4000 years, dating back to the pharaonic period, and have widely proliferated with the appearance of synthetic surfactants a century ago. Synthetic surfactants used to make macro-micro-nano-emulsions and foams are used in laundry and detergency, cosmetics and pharmaceuticals, food conditioning, emulsified paints, explosives, enhanced oil recovery, wastewater treatment, etc. The introduction of a multivariable approach such as the normalized hydrophilic–lipophilic deviation (HLD _N_) and of specific structures, tailored with an intramolecular extension to increase solubilization (the so-called extended surfactants), makes it possible to improve the results and performance in surfactant–oil–water systems and their applications. This article aims to present an up-to-date overview of extended surfactants. We first present an introduction regarding physicochemical formulation and its relationship with performance. The second part deals with the importance of HLD _N_ to make a straightforward classification according to the type of surfactants and how formulation parameters can be used to understand the need for an extension of the molecule reach into the oil and water phases. Then, extended surfactant characteristics and strategies to increase performance are outlined. Finally, two specific applications, i.e., drilling fluids and crude oil dewatering, are described.

## 1. Introduction

The synthesis of surfactants for the solubilization of complex oils, including crude oils and polar oils, has been widely researched since the late 1970s in universities and industrial settings [1,2]. After petroleum sulfonates were introduced [3,4,5], it was necessary to develop high-performance surfactants with reasonable production costs to advance surfactant flooding processes. Henceforth, ethoxylated oleyl sulfonates (EOS) were introduced [6,7] as the first surfactants that presented an extension from the usual alkyl chain—sulfate head surfactants. The first EOS surfactants had three ethylene oxide groups in their structure, which allowed a somewhat higher performance, although still with the need of cosurfactants to achieve high solubilizations and low IFT [6]. They were tried with long-chain hydrocarbons as the oil phase, e.g., hexadecane and paraffin oils [6,7]. Then, an additional intramolecular extension with four PO was added to sulfate surfactants [8], which allowed attaining high performance without the need for cosurfactant alcohol. This was a significant advancement in the simplification of the system, using an innovative solution, i.e., including a PO-EO intramolecular extension to generate the polarity transition inside the molecule that produced more interactions with the oil and aqueous phases. A summary and classification of the sulfate head extended surfactants developed since 1992 with the reported surfactant classification parameter (SCP_N_) [9,10,11] is presented in Table 1. Nevertheless, other molecules have been developed with different types of polar heads (carboxylate, xylitol, glucose derived, ethoxylated nonionic), as summarized in the 2019 review [2].

The need for the solubilization of polar oils (triglycerides, oleic acids), which present very low solubilization with conventional surfactants, led to trying these new molecules. The polar oils tested included perchloroethylene oil [12] and ethyl oleate [13] with a (surprising at the time) very high performance. Miñana et al. [13] also tried the solubilization of triglycerides (which at the time was practically zero with conventional surfactants, even with cosurfactant alcohols) with C_12_POnEO_2_SO_4_ (*n* = 6, 10 or 14), showing for the first time high solubilization of these bulky polar oils. In the same year, Aoudia et al. [14] achieved high performance with crude oils and a very long PO = 15 extension in a C_14_PO_15_SO_4_ surfactant. These first state-of-the-art extended surfactants synthetized and proved for high solubilization of polar oils allowed a progressive advance of research [15,16]. Nevertheless, it was not until the early 2000s that new molecules were developed at FIRP Lab. [17,18,19,20], including surfactants with a carboxylate head and others derived from triglycerides and natural sugars [21,22]. These first trends concerning new extended surfactants for the high solubilization of polar oils allowed the progressive advance of research [2,20,23].

Several other surfactants that were tried since 1995 were used in different applications, including EOR and the solubilization of polar oils [24,25,26,27,28,29,30], but also other not-so-conventional applications, such as drilling fluids [31,32,33] and crude oil dewatering [34,35,36].

In 2005, the first review on extended surfactants was published [1], indicating the rationale for developing such intramolecular PO extension and its relationship with solubilization. In 2019, a thorough update on all the works up to date on the field was published, including some strategies to increase the solubilization of polar oils and performance [2]. In 2021, two extensive reviews on some applications of extended surfactants were published, one focused on EOR applications [37] and another on vegetable oil extraction [38].

**Table 1:** Molecular structure and classification of sulfate head extended surfactants according to its normalized characteristic parameter (SCP_N_) [2].

Extended Surfactant ^1^	σ	k	SCP_N_ = σ/k *	Author and Year	Ref.
S/12/6/2/SO_4_	−1.43	0.075	−19.1	Miñana-Perez, 1995	[13]
S/12/10/2/SO_4_	−0.3	0.11	−2.7	Miñana-Perez, 1995	[13]
S/12/14/2/SO_4_	1.21	0.16	7.6	Miñana-Perez, 1995	[13]
A/14−15/8/0/SO_4_	0.16	0.13	1.2	Witthayapanyanon, 2006	[30]
A/10/18/2/SO_4_	0.57	0.053	10.8	Do, 2009	[39]
A/14−15/4/0/SO_4_	−0.18	0.11	−1.6	Velásquez, 2010	[24]
A/16−17/4/0/SO_4_	−0.29	0.11	−2.6	Velásquez, 2010	[24]
A/12−13/8/0/SO_4_	−0.52	0.08	−6.5	Velásquez, 2010	[24]
A/12−13/4/0/SO_4_	−0.98	0.11	−8.9	Velásquez, 2010	[24]
Chen/8/9/3/SO_4_	−0.39	0.17	−2.3	Chen, 2019	[40]
A/12−13/4/0/SO_4_	−1.55	0.049	−31.6	Wang, 2019	[41]
He/13/2/0/SO_4_	−1.8	0.056	−32.1	He, 2019	[42]
A/10/4/0/SO_4_	−2.24	0.053	−42.3	Phaodee, 2020	[43]

^1^ Nomenclature: A: Alfoterra, S: Seppic, Chen and He are the first authors of the papers where these surfactants were synthetized. A/10/18/2/ SO_4_ stands for Alfoterra/C_10_/PO_18_/O_2_/SO_4_. This is the same nomenclature as [2]. * SCP_N_ is the surfactant classification parameter. Higher SCP_N_ indicates a more important lipophilic part of the molecule (hydrocarbon tail and PO extension); a lower SCP_N_ (more negative) indicates a more important hydrophilic head contribution.

In the present work, we update the 2005 review indicating the need for developing such specifically tailored molecules and their relationship with HLD_N_, and two recently divulgated applications, e.g., the use of extended surfactants in drilling fluids and in crude oil dewatering. This study is divided into two parts. The first part presents a review of the basic concepts necessary to understand how the development of extended surfactants is derived from physicochemical formulation principles, including:

1. Formulation concepts,

2. The generalized HLD expression for the optimum formulation,

3. Performance issues (introducing strategies to attain high performance changing variables in the normalized HLD_N_, and how HLD_N_ can be used for surfactant mixtures, which leads to Section 4),

4. Lipophilic and hydrophilic linkers and extended surfactants (the particular characteristics of extended surfactants and the rationale for their development are reviewed, in addition to the use of HLD_N_ with extended surfactant mixtures).

In the second part, we describe some unconventional cases in a real application and how to solve them:

5. Extended surfactants for drilling fluids applications

6. Improved performance and robustness in crude oil dewatering and dehydration

We finish by indicating that examples can be found in multiple applications of extended surfactants. Therefore, contributions regarding new types of biobased extended surfactants [17,18,22] that could comply with new environmental regulations can help to further advance knowledge in this field.

## 2. Historical Introduction on Formulation Concepts

It can be said that a century ago, the so-called Bancroft’s rule and its related research and development discussions [44,45,46] were the first attempt to attain a generalized practical approach for surfactant–oil–water (SOW) systems. However, it was only in the late 1950s that two researchers from industry tried to improve the practical aspects related to SOW systems.

Griffin [47] introduced the so-called hydrophilic–lipophilic balance parameter, called HLB. Some time later [48], he proposed several numerical expressions to estimate the HLB number as a function of the chemical structure of nonionic surfactants, e.g., 20% of the polyoxyethylene weight for an ethoxylated alcohol. HLB was related to the surfactant effect and was thus the first numerical scale that could help compare cases and averaging effects. Even though it did not take into account the effect of other variables, it was the unique numerical criterion for 25 years because it was an extremely simple concept. Thus, it is still currently used as approximate information for people in the industry who do not require high accuracy in formulation work [49].

**Figure 1:**
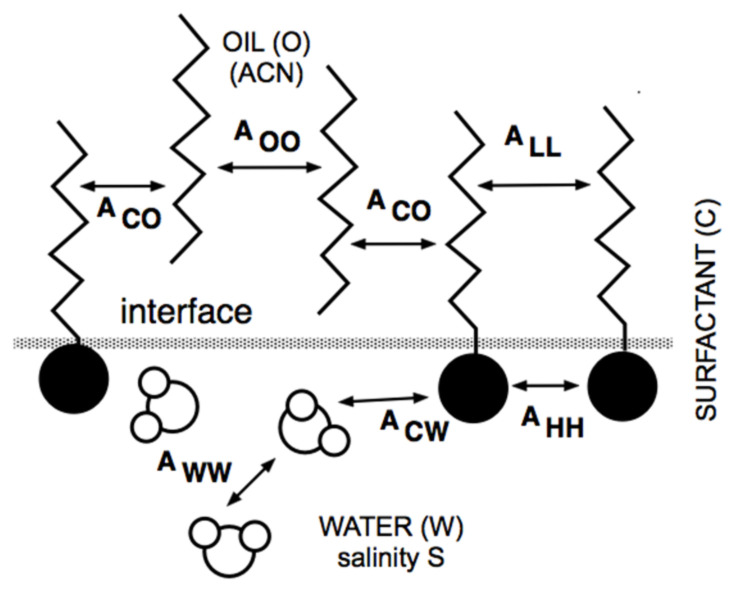
Interaction of surfactant, oil and water molecules close to interface according to Winsor’s scheme.

At the same time, but in a completely different research area, Winsor [50] proposed a complex model based on the ratio R of interactions between the surfactant adsorbed at the interface and the neighboring oil and water molecules on both sides of it, indicated explicitly as A_CO_ and A_CW_ in Figure 1.

The original model R = A_CO_/A_CW_ was made more realistic by introducing the self-interactions between the surfactant, oil and water molecule as references, each in separated terms [51,52].

Thus, this more precise approach resulted in a new definition of the interaction ratio as R = (A_CO_ − A_OO_ − A_LL_)/(A_CW_ − A_WW_ − A_HH_), which is not discussed here because it is out of the scope of this review. Nevertheless, and as has been explained elsewhere [11,53,54,55], it must be noted that the effective interaction between the surfactant and the oil molecules, i.e., the numerator of R, tends to decrease when the oil ACN increases. This happens because A_OO_ (between two *n*-alkane molecules) increases, in general, faster than A_CO_ (between the surfactant tail and an oil molecule), while the other terms are unaltered. In what follows, R is taken as (A_CO_ − A_OO_)/A_CW_ for simplicity.

### The Unidimensional Scan of a Formulation Variable

In his research in the late 1940s, Winsor proposed a basic method to study a surfactant–oil–water (SOW) system by determining its phase behavior versus a continuous variation (called a scan) of a variable susceptible to alter at least one of the interactions [51]. In simple systems, these variables typically were the oil nature (e.g., the *n*-alkane carbon number ACN), the water NaCl salinity (S), the surfactant and co-surfactant type (head or tail nature), the temperature, and eventually the pressure [56,57,58], which is an essential variable in a petroleum reservoir.

**Figure 2:**
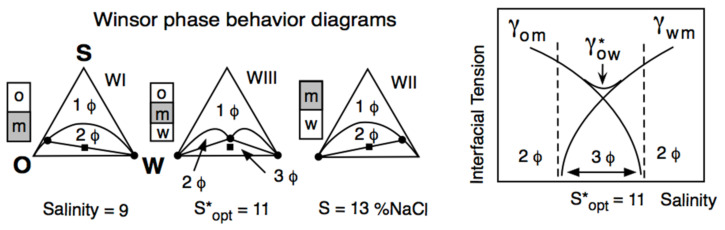
Variation of the interfacial tension (right) and of the phase behavior in a ternary diagram (left) along a formulation unidimensional scan (here, the salinity of the aqueous phase S). S* and γ* are the salinity and the interfacial tension at optimum formulation, respectively.

Winsor reported that a three-phase behavior (central diagram in Figure 2 left part) was occurring exactly at R = 1 and was associated with a low interfacial tension, indicated as γ* in Figure 2, right part.

In Figure 2, the water salinity is the scanned variable and R increases when the salinity increases because the surfactant interaction with water (A_CW_ in R denominator) tends to decrease. At the R = 1 situation (S = 11 wt% NaCl salinity in Figure 2), the surfactant interaction with water exactly compensates its interaction with the oil. In all the test tubes shown, “m” indicates the phase that contains most of the surfactant, generally with a turbid aspect because of the light backscattering produced by some structure, such as a swollen micellar aggregation. The other phases are transparent, with a very low surfactant concentration, typically close to the critical micelle concentration. At S = 9, most of the surfactant is in water, and the system exhibits a so-called WI phase behavior with an excess oil phase, while at S = 13, it is the opposite WII case.

The intermediate case is called WIII phase behavior (3Φ), with a middle phase containing a structure that has been called a bicontinuous microemulsion [59]. This can be a misleading term (which was not accepted by Winsor) since it is not an emulsion but a structure with an average zero curvature surface [60,61].

More information on the change of the Winsor diagrams along a single variable scan can be found elsewhere [62,63,64].

The very specific property shown in Figure 2, i.e., the coincidence of a three-phase behavior and a minimum interfacial tension, was rediscovered in 1975 by industrial and academic researchers dedicated to developing an enhanced oil recovery technique (called EOR) by injecting a surfactant solution in a petroleum reservoir. The formulation corresponding to the middle of the three-phase behavior zone was called “optimum”, i.e., optimum salinity S* in Figure 2. This happens because it produces the very low interfacial tension minimum between brine and petroleum, eliminating the capillary blocking occurring in a porous reservoir after the conventional water flooding secondary recovery [65].

**Figure 3:**
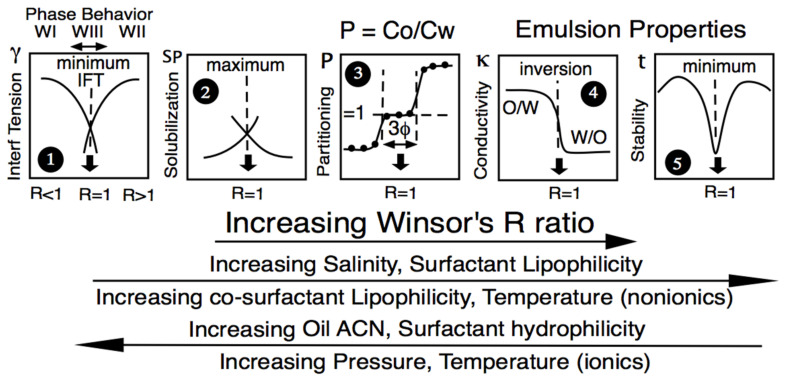
Variation of some SOW equilibrium or emulsified properties along a formulation scan, with R increasing from left to right according to the variable changes indicated below in the direction of the arrows.

At the so-called optimum formulation of a scan, whatever the formulation variable used to alter the interactions—not only water salinity but also oil ACN, temperature; the modification of surfactant hydrophilicity-lipophilicity (as the tail length or the head group, mainly the ethoxylation number EON for nonionics); co-surfactant type and concentration, etc.—many specific events were found to occur in the 1970s and later, as schematically indicated in Figure 3. First, the three-phase behavior with a microemulsion in equilibrium with excess water and oil phases containing a very low surfactant concentration was close to the CMC. This minimum interfacial tension, often below 0.001 mN/m (plot #1), matches a maximum solubilization in plot #2 according to the Chun Hun model [66]. Other specific properties were found in non-equilibrated systems, such as the emulsion phase inversion (plot #4), particularly with a temperature scan [67], or the minimum emulsion stability (plot #5) [68,69,70,71], later related to a minimum interfacial rheology [36,72,73]. The central plot #3 in Figure 3 indicates that the surfactant partitioning between the two excess phases in an optimum WIII system is in exact unity. This property was first reported in the early studies on EOR with commercial anionic surfactants [5,74], being important from the theoretical point of view, even though it does not really matter in practice.

Let us call µ the surfactant chemical potential and µ^S^ the standard chemical potential in some fixed conditions. The classical expression for the variation of chemical potential with the surfactant activity is:

µ_O_ = µ_O_^S^ + RT LnC_O_ in oil and µ_W_ = µ_W_^S^ + RT LnC_W_ in water (1)

where Co and Cw indicates the effective concentration, which is basically the same as the activity at low concentration. At equilibrium, the chemical potential of the surfactant in oil and water is the same, i.e., µ_O_ = µ_W_. If this equilibrium happens at optimum formulation with a three-phase system where the concentrations in excess phases are equal (C_O_ = C_W_), then the standard chemical potentials are equal.

µ_W_^S^ = µ_O_^S^ or Δµ^S^ = µ_W_^S^ − µ_O_^S^ = 0 = SAD (2)

In this relation, the difference in standard chemical potential Δµ^S^ is called the surfactant affinity difference (SAD), using the term “affinity” for the negative of the chemical potential.

Of course, since both standard chemical potentials depend on the system formulation variables, SAD is a function of all the variables that alter the interactions of the surfactant with oil or water, such as the water salinity, oil nature (ACN), interfacial surfactant and co-surfactant head and tail characteristics, as well as temperature and pressure.

If all these variable effects are supposed to be independent, they can be numerically characterized. Moreover, the R = A_CO_/A_CW_ = 1 relation that indicates optimum formulation can equally be written as a zero difference D = A_CO_ − A_CW_ [71], which is now expressed as SAD = Δµ^S^ = 0 at optimum. If SAD > 0, the affinity of the surfactant for oil is larger, and R > 1, and vice versa.

In the evolution of this SAD numerical equation, this difference was first written as a variation in a unidimensional scan, e.g., ΔS in a salinity scan, ΔACN for an oil scan, ΔEON in an ethoxylated surfactant head size scan, ΔT in a temperature scan, etc.

Since the chemical potential µ has an energy dimension, the SAD = 0 equation was divided by RT to make it dimensionless. It was called the hydrophilic–lipophilic deviation (or difference) and abbreviated as HLD [75]. The optimum formulation was thus expressed as HLD = SAD/RT = 0.

## 3. Multivariable Scans and Generalized HLD Expression for Optimum Formulation

The generalized Figure 3 indicates that the increase in some formulation variables produces a property transition opposite to that generated by the increase or decrease of other variables. This means that two variables are likely to compensate their effects if they are both properly changed. For instance, according to Figure 3, an increase in salinity increases R (thus increasing HLD); it thus produces a phase behavior WI > WIII > WII transition, while an increase in ACN produces the opposite WII > WIII > WI.

**Figure 4:**
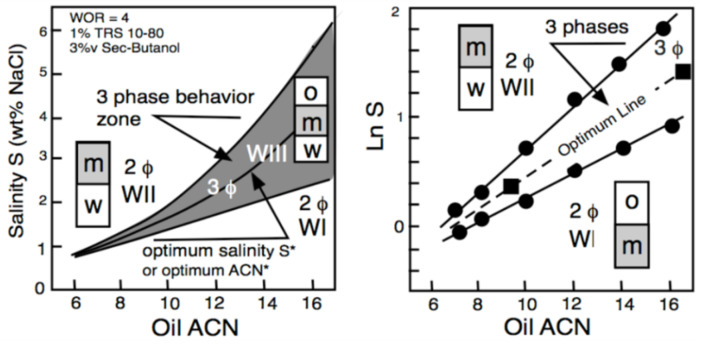
Bidimensional (water salinity and oil ACN) formulation scan [5]. S* is the salinity at optimum ormulation.

Figure 4 displays the phase behavior transitions in the two-dimensional S-ACN space when all other variables are constant. In the left plot, numerical values are indicated in the ordinate for salinity and in abscissa for ACN. The gray area indicates the three-phase zone (WIII). A vertical variation of salinity at the ACN constant corresponds to Figure 2 salinity scan, while a horizontal variation of ACN at constant salinity is an ACN scan. The line at the center of the three-phase zone is the optimum formulation line in a bidimensional scan; it corresponds to the optimum ACN* at constant salinity and to the optimum S* at constant ACN. Hence, an increase in salinity results in the transition WI > WIII > WII, while an increase in ACN does the opposite. Figure 4 corresponds to a typical system for EOR, containing 1 wt% of a commercial alkylbenzene sulfonate with 3 vol% of 2-butanol co-surfactant at a fixed temperature and pressure. It can be seen in Figure 4 (right plot) that the optimum formulation line at the center of the three-phase zone becomes exactly a straight line if the LnS scale is used in the ordinate.

Consequently, the equation of the optimum formulation line in this LnS-ACN space may be written as LnS = K_A_ ACN + CST, where the constant term (CST) depends on the other variables that do not change, i.e., the surfactant and cosurfactant, the temperature and the pressure. Such a bidimensional variation of both oil ACN and water salinity (for instance, between the two square dots located on the optimum line) may be expressed as ΔHLD = ΔLnS − K_A_ ΔACN = 0 at all other constant variables.

Numerous studies from various laboratories in the past 40 years [24,54,76,77,78,79,80] indicated that this linear relationship is essentially valid (at least over some range of ACN of about 10 units for all ionic surfactants). However, the ACN coefficient K_A_ value was found to depend on the surfactant, particularly its head group. It is 0.16 for alkylbenzene sulfonate, 0.10 for *n*-alkyl sulfate or carboxylate, 0.17 for the dihexylsulfosuccinate, and 0.20 for alkyl trimethyl ammonium salts. For extended surfactants with several blocks, such as the alkyl polypropoxy ethoxy sulfates or carboxylates, this K_A_ coefficient is particularly low, i.e., 0.05 to 0.10, and depends on the exact structure—in particular, the alkoxylated central block size [2].

A slight departure from linearity with LnS has been found for complex head groups, mainly when there is an internal or external mixture of ionic and nonionic parts [6,14,81]. A linear relation between S (instead of LnS) and ACN generally better matches ethoxylated nonionic surfactant [54,82]. However, the effect is much weaker than for ionic species and a partitioning inconvenience occurs with commercial surfactants, affecting the numerical data accuracy.

This double scan technique with an exact compensation of two opposite effects was introduced by the University of Texas group in 1977 and was later applied to many different pairs of variables susceptible to altering the interactions of the surfactant with both oil and water. The equations summarized in Table 2 indicate numerical results from a double scan change involving two variables, i.e., ΔHLD = 0, from our data and other publications cited in the two previous paragraphs.

### 3.1. The Normalized Hydrophilic Lipophilic Deviation (HLD_N_) Equation

The previous data comes from many different experiments. It should be noted that the numerical values of the equivalence of the change between two variables are arbitrarily written as K_1_ ΔV_1_ − K_2_ ΔV_2_ = 0, which could be systematically written as ΔV_1_ = K_21_ ΔV_2_ with the coefficient K_21_ = K_2_/K_1_, as the linear equivalence in the ΔHLD double change.

The proper variable to make a comparison between different cases is to have the same change, which has been recently taken as a unit change in ACN, which is the same in all the cases [9,11]. Consequently, the previous list of equations on ΔHLD = 0 will be divided by the coefficient before ACN, so that in the “normalized” expression, it would become −1 as in Table 3 ΔHLD_Ni_ Equations (1)–(3). The minus sign is explained by the fact that an increase in ACN tends to decrease the numerator of R, thus reducing the HLD.

Thus, all the previous equations will be written as follows with the properly evaluated or calculated K_i_ coefficient before any V_i_ variable. The normalized equation implies ACN and at least one V_i_ variable to produce the double scan compensation but can include 3, 4, 5, etc.

ΔHLD_N_ = 1 − 1 = 0 = − ΔACN + ∑ ± K_i_ ΔV_i_ with i = 2, 3, 4 etc. (3)

If the double change does not include a unit variation of ΔACN, it should include variations of (at least) two other variables that are equivalent to ΔACN = ±1, as in the ΔHLD_Ni_ equations numbers 4, 5, 7 in the following list, which indicate the proper coefficient K_i_ before all formulation variables.

The data displayed in Table 3 deserve some specific comments. When a − ΔACN term appears in one of the equations, a positive effect of another term indicates that the corresponding variable change increases the HLD. It means that it is increasing the interaction of the surfactant with oil or diminishing its interaction with water by an amount equivalent to a negative unit of ACN. Therefore, from the previous Table 3 data, diminishing the ACN by one unit has the same effect as adding 0.44 carbon atoms in the surfactant *n*-alkyl tail (SAT) or adding 0.83 propylene oxide group (PON) in the intermediate part of an alkyl polypropoxy extended surfactant, or removing 0.24 ethylene oxide group from the head (EON) of an ethoxylated alcohol.

Transforming expression (4) to a differential equation level and taking into account that the first partial derivatives are constant (over some range) [10,11]

dHLD_N_ = 0 = −dACN + ∑ ± K_i_ ΔdV_i_ with i = 2, 3, 4 etc. (4)

and integrating it

HLD_N_ = 0 = −(ACN−ACNref) + ∑ ± Ki (Vi − Vi ref) with i = 2, 3, 4 etc. (5)

Including an integration constant which is zero when all the variables are at a reference in which there is an optimum formulation.

Using only four variables to describe a simple system, i.e., surfactant, oil, water and temperature, the equation HLD_N_ = 0 at optimum formulation can be written as follows for ionic and nonionic surfactants [9]:

HLD_N_ = K_S_ (LnS/S_ref_) − (ACN−ACN_ref_) − K_TIO_ (T-T_ref_) + SCP_N_ − SCP_Nref_ (ionic) (6)

HLD_N_ = K_S_ (S-S_ref_) − (ACN−ACN_ref_) + K_TNI_ (T-T_ref_) + SCP_N_ − SCP_Nref_ (nonionic) (7)

In the past few years and with or without clear explanations, the references have been taken as follows. S_ref_ = 1, because ionic surfactants are salts that thus provide a minimum salinity or equivalent salinity in wt% NaCl, which is in general much smaller than 1 wt% [83]. What is favorable is that, consequently, LnS_ref_ becomes zero in the ionic surfactant equation. A Sref = 0 often provides the reference for nonionic surfactants, but it is unfortunately different from the ionic case. Thus, it might be more logical to use S_ref_ = 1 in all the cases, the term K_S_ S_ref_ being almost negligible in most cases. ACN_ref_ = 0 also simplifies the equations, even if it is not a very logical choice, since it corresponds to no carbon atom in the *n*-alkane, i.e., something which does not exist. However, an equivalent EACN = 0 was found to be some approximation for benzene [84]. The temperature reference is often taken as 25 °C, but also at T = 0 °C, which simplifies the equation. This occurs even though it often corresponds to a solid-state of oil. In this simplified equation, there is no particular term for the pressure effect nor for a co-surfactant, initially called f(A) [76]; however, if this is so, the references would be the atmospheric pressure and the absence of co-surfactant.

### 3.2. The Normalized Surfactant Characteristic Parameter (SCP_N_)

The SCP_N_ abbreviation means the normalized surfactant contribution term [9,11], which, for several reasons explained below, has been a source of confusion over the past 40 years. First of all, it was referred to with names depending on the experiments. In the early 1970s studies [4], it was called N_min_ because it corresponded to the minimum tension in an ACN scan. The term hydrocarbon “preference” was introduced at the same time [4,85]; thus, the PACN name (i.e., preferred ACN in an ACN scan) was also used very early. This happened especially with a more detailed definition as EPACNUS for “extrapolated PACN at unit salinity and no-alcohol” in the original publications of a multivariable correlation for optimum formulation, also called σ/K_A_ for ionic and β/K_A_ for nonionics in the literature [5,76,86], including a very comprehensive recent review [55]. To avoid confusion, the SCP_ref_ term is always taken as zero. However, it could be a misleading value when it is related to a characteristic surfactant curvature since it depends on many aspects other than the surfactant [10,87].

According to data published for very pure systems containing single oligomer ethoxylated alcohols [88], the normalized equation for the optimum formulation was found to be as follows for S in wt%NaCl and T in °C, for a range around EON~7 and T~30 to 50 °C with notable variations of K_TNI_ with EON and T [89]:

HLD_N_ = −ACN + 0.87 S + 0.03 T + SCP_N_ = 0 (8)

HLD_N_ = −ACN + 0.87 S + 0.03 T − 6.67 EON + 2.26 SAT + 13 = 0 (9)

This means that the last term (13 ± 1) comprises all the references (including SCPN_ref_ taken as zero or not), plus the constant integration value, so that when the data of an optimum system (its ACN, S, T, EON, and SAT) are entered, then the equation HLD_N_ = 0 is valid.

Therefore, if any reference is changed, then the last term will also change. Nevertheless, what is very important to understand is that the term SCP_N_ changes with the surfactant, i.e., it increases by 2.26 when a carbon atom is added to its *n*-alkyl tail, and it decreases by 6.67 when an EON group is added to its polyethoxylated head). Then, its value depends on the references for all the variables, which is the reason why it is not a surfactant characteristic.

HLD_N_ = 0 equation makes it possible to numerically express the conditions for an optimum formulation in the same scale (ΔACN unit change). It thus allows to make comparisons between the corresponding SCP_N_ values for various surfactants, and it also makes it possible to calculate the values of mixtures according to

HLD_Nmix_ = ∑X_i_ HLD_Ni_ or SCP_Nmix_ = ∑Xi SCP_Ni_ (10)

With X indicating the molar fraction at the interface, which is often approximated as the weight fraction in the system, although such linearity could be erroneous in the presence of partitioning of the different species, as in many commercial surfactants. [75,84,90,91].

## 4. Performance Issues

Now that the optimum formulation to produce a minimum tension or maximum solubilization is known to happen at HLD_N_ = 0, the second practical question is to understand how low the minimum tension is or how high the maximum solubilization is.

Two cases must be considered to answer this question, which are easier to distinguish using the unit R ratio situation to express the equal interactions at optimum formulation.

### 4.1. Attaining a High Performance through a Double Formulation Change at Both Sides of the Interface

In the first case, the double change occurs on the two sides of the interface, for instance, an increase in salinity that diminishes the denominator A_CW_, and an increase in ACN which decreases the numerator (A_CO_-A_OO_) in the same amount, according to the empirical equation ΔHLD_1_ = 0, i.e., ΔLnS = 0.16 ΔACN for a sulfonate. It is akin to saying that the double change passed, for instance, from R = 1 = 5/5 to R = 1 = 2/2. In practice, this means that the new optimum formulation will still produce the minimum tension in an ACN or a LnS scan, but that this minimum will be higher, i.e., worse as far as the EOR application is concerned.

The double scan is produced by increasing the interaction of the head of a nonionic surfactant with water by adding some ethylene oxide group (increasing EON). To compensate it on the other side, the interaction with oil is increased by adding more carbon atoms in the surfactant *n*-alkyl tail (increasing SAT). Then, the empirical equation for the double change will be according to the experimental data ΔHLD_4_ = 0. i.e., ΔEON = 0.33 ΔSAT.

This means that three more carbon atoms in the surfactant linear tail increase the interaction with the oil in the same way as one more ethylene oxide group in the head with the water. This double change will thus improve the interactions on both sides and, consequently, improve the surfactant performance. It has been experimentally shown [89] that in a system containing an extremely pure ethoxylated *n*-alcohol, such a double increase reduced the interfacial tension by almost 20 times. From the enhanced oil recovery point of view, this represents a significant performance improvement, i.e., an increase in 1.2 units of the “performance index,” which is the negative of the decimal logarithm of the minimum interfacial tension (PERFIND = −logγ_min_). As far as the Winsor approach is concerned, the compensating double increase of EON and SAT is similar to a change from R = 5/5 to R = 20/20.

### 4.2. Attaining a High Performance through a Double Formulation Change at One Side of the Interface

The second case of double change in interactions to keep an optimum formulation situation is when the two variations occur on the same side of the interface. For instance, when the surfactant head interaction with water is increased by adding EON groups and (equally) decreased by adding more salt in the water phase. Such a case has been experimentally shown in the ΔHLD_5_ = 0 equation, i.e., ΔEON = −0.13 ΔS [82] with the NaCl salinity S in mole/liter in such an equation).

A similar double change can be carried out on the oil side by increasing the interaction. This is performed by adding carbon atoms in the surfactant tail (SAT change increasing A_CO_), thus increasing the numerator of R, then by increasing the alkane length (ACN) that increases more A_OO_ than A_CO,_ and thus decreases the numerator (A_CO_-A_OO_) to return it to the original value. The experimentally found equivalence was written as ΔHLD_6_ = 0; i.e., 2.25 ΔSAT = ΔACN.

In this second type of double change, the interactions on one side are altered twice in opposite directions, while on the other side, the interactions are unchanged. Consequently, this double change does not alter the numerator nor the denominator of R, and the performance is expected to remain unchanged. Experimental data corroborated this phenomenon in the literature, mainly using solubilization as the performance criterion [92].

Consequently, the kind of double change method to be used to improve the performance is the first one, i.e., the increase in interactions of the surfactant on both sides of the interface.

Increasing the interactions of the surfactant on the water side is relatively easy for different reasons. A polar head with more than one charge can be used in ionic surfactants, e.g., taking a phosphate anion or a double head such as in gemini or zwitterionic surfactants. Bigger heads such as sulfosuccinate, or mixed ionic/nonionic combinations as polyethoxysulfate easily provide more hydrophilic affinity. Additionally, it is worth noting that polyethoxylated chains are partially compatible with most oil phases, including hydrocarbons. They can thus be quite long, with many interactions with water.

It can be said that the main challenge to improve the performance is to increase the surfactant tail interaction with the oil phase while keeping enough compatibility with the water phase to increase its solubility and avoid its precipitation, particularly in the cases where it has to be introduced in practice as an aqueous solution such as in EOR. Adding co-surfactants such as short alcohols—particularly species that do not alter the formulation, such as sec-butanol, as was proposed in the 1970s and 1980s [65]—that tend to accumulate at the interface, reduce the probability of precipitation. However, in most cases, it also reduces the performance because it diminishes the surfactant interfacial adsorption.

### 4.3. Additional Strategies to Increase Performance

During the last 40 years, many studies have been carried out with hydrocarbon oils, including crudes. These studies have shown that the longest *n*-alkyl tail that can be used to avoid problems is hexadecyl (C16). They have also shown that the performance could be increased by modifying the molecular structure organization around the interfacial layer in some appropriate way, maintaining a dense adsorption and avoiding incompatible contact [93].

Different methods can achieve this. The first one was found to be the branching of the tail [81,94], whose increase eliminates the precipitation problem but also produces other interesting changes. These were found by carrying out ACN scans with differently branched *n*-alkyl benzene sulfonate oligomers, e.g., having the aromatic ring in different positions of the same *n*-hexadecyl tail [95,96]. In the maximum branching structure, when the benzene sulfonate hydrophilic group was at the center of the tail (8ΦC_16_SO_3_Na), the optimum ACN in the scan was the highest value (called N_min_ or PACN at this time but corresponding to what is today called the SCP_N_); i.e., the surfactant was more lipophilic. Nevertheless, even if it was more lipophilic, it was also found to be more compatible with water. This was because the two C8 branches are sticking together; therefore, the lipophilic length incompatible with water is thus much shorter.

Additionally, this 8ΦC16SO_3_Na oligomer was not found to be the best nor the worst structure as far as the performance was concerned. It occurred that the minimum tension value was encountered for the 5ΦC_16_SO_3_Na species, i.e., with two different branches in the tail, one with 10 carbon atoms and the other with five. This noteworthy information indicated that both the formulation and the performance depended on the interfacial structure in different ways.

**Figure 5:**
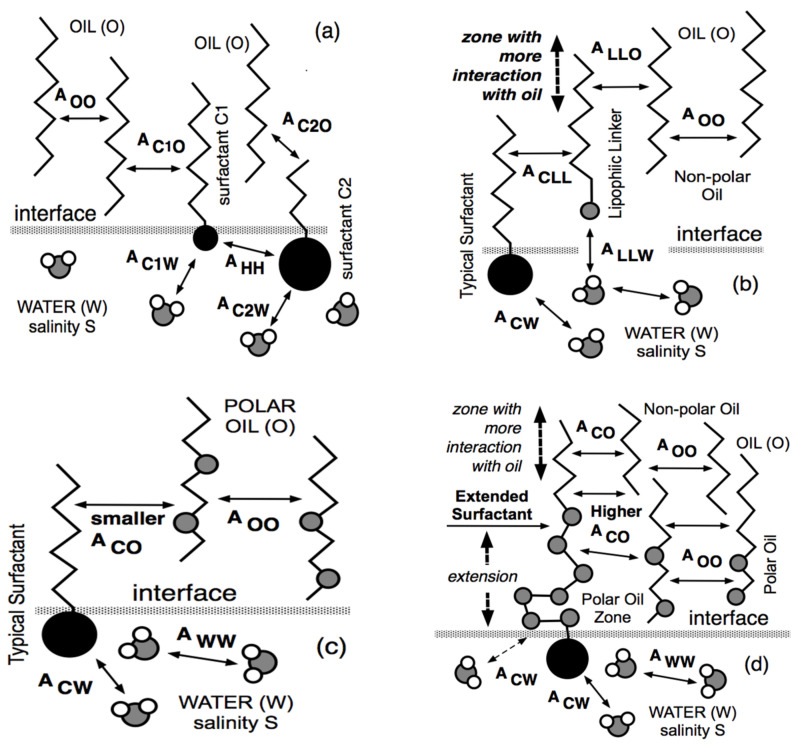
Interfacial arrangements (**a**) with two ordinary surfactants C1 and C1, (**b**) with a surfactant and a lipophilic linker, (**c**) with an ordinary surfactant and a polar oil, and (**d**) with an extended surfactant and a polar oil.

The second method which was found to improve the performance was to mix two surfactants, typically a lipophilic one called C1 in Figure 5a with a small head and a long tail, with a hydrophilic one called C2 with a small tail and a big head. Note that in Figure 5, the small polar parts (such as oxygen atoms) are indicated in gray. As shown in Figure 5a, most of C1 is in oil, while most of C2 is in water, and as a combination, the mixture has a more significant interaction both with oil (because of the long C1 tail) and with water (because of C2 big head). Mixtures of surfactant were proposed in the 1970s [5,97,98,99] and mixing rules were established with the multivariable correlations dealing with the attainment of the optimum formulation [86,100].

However, the mixture of quite different surfactants presented a serious problem due to a different partitioning of the species, with a large proportion of the lipophilic species inside the oil phase and the hydrophilic inside the water phase. Since the interfacial effect depends on what is at the interface, this partitioning phenomenon loses part of the incorporated surfactant, sometimes a very high percentage, e.g., 80%.

## 5. Lipophilic and Hydrophilic Linkers, and Extended Surfactants

### 5.1. The Lipophilic Linker

Since the main difficulty was to increase the interaction on the oil side, a special C1 highly lipophilic amphiphile was used to replace the C1 surfactant, as indicated in Figure 5b, while the C2 surfactant was only slightly hydrophilic, and thus was likely to mainly go to the interface. This C1 species was typically an *n*-decanol or dodecanol that was not really a surfactant adsorbed at interface but rather a polar oil segregated in the oil phase close to the interface because of the affinity of the OH end group with water [101].

This kind of substance was called a lipophilic linker [102,103,104,105,106] because it was not a co-surfactant (since it was not adsorbed at the interface) but was a species of the oil phase staying close to the interface, as shown in Figure 5b. As a consequence of its location, the lipophilic linker was more or less sticking to the surfactant tail and somehow extending it further in the oil phase. Therefore, it was increasing the interaction on the oil side, and thus improving the performance.

### 5.2. The Hydrophilic Linker

It was suggested that a similar intermediate be placed on the other side of the interface between the surfactant head and the water and was called a hydrophilic linker [107,108]. Its benefit was, however, much less significant, mainly because it was not producing a much higher penetration of the surfactant head group into water.

Moreover, it was known that the solubilization of a polar oil with an ordinary surfactant having a head group and an alkyl tail, as indicated in Figure 5c, was not very good because of a lack of perfect matching of the hydrocarbon tail with the polar oil. This is why adding a lipophilic linker in the oil phase close to the interface produced an improvement of the interaction with polar oil.

Nevertheless, the lipophilic linker was actually an oil phase component; a significant amount of it was lost in the oil bulk far from the interface, and thus partitioning was even worse with a polar oil. It was thus necessary to find a means to cumulate the favorable effects and avoid the unwanted ones.

### 5.3. The Extended Surfactant with an Intramolecular PO Extension

The idea developed in the 1990s [13,15,77,109] was to fasten together the rather hydrophilic surfactant and the lipophilic linker, producing the same effect as the mixture without losing a part of the lipophilic linker deviating from the interface. Since the lipophilic linker was an amphiphile with a small hydrophilic part located close to the interface, the single structure shown in Figure 5d to imitate the mixture situation is a so-called “extended” surfactant. It contains a polar head located in water, then an intermediate slightly polar zone in the oil phase close to the interface, and finally, the surfactant classical hydrocarbon tail. Figure 5d shows that the slightly polar intermediate extension is bent and interacts with the water molecules, producing more A_CW_ interaction.

The central extension was selected to be a polypropylene oxide (PO) because it was neatly lipophilic and was thus located on the oil side of the interface with good interaction with polar oil molecules. It also perfectly plays its main role of displacing the alkyl part of the tail further away from the interface, as shown in Figure 5d, without the usual precipitation problem at increasing the tail size. It is worth noting that our original extended surfactants, e.g., C_12_PO_N_EO_2_SO_4_^-^Na^+^, had a much longer intermediate (N up 14) than the alkoxylated surfactants available at this time, which had very few units only. It should be noted that a 10-unit PO chain is about three times longer than a C12 *n*-alkyl group, so that the actual tail extension is considerable, even if it is not completely perpendicular to the interface [20,110].

The PO chain is in the zone where the polar oil molecules are segregated, while the alkyl hydrocarbon part of the tail could be far away from the interface where most of the oil could be the non-polar one. This was an extra matching situation between the surfactant tail and the oil phase containing polar molecules. Sometimes, a very short ethoxylation (1–2 groups) is placed at the end of the PO chain, not really as a hydrophilic linker part, but rather because it makes an easier addition of the sulfate or other hydrophilic group [17,18,19,21,22].

The improving performance with polar oil was reported first by Miñana [13,77], particularly in mixtures of extended surfactants with ordinary species that were not solubilizing at all triglyceride vegetable oils. Most research carried out in the following years used different anionic and nonionic heads sometimes connected by two EO groups to the propoxylated extension [111].

### 5.4. The Increased Performance of Extended Surfactant Systems with Polar Oils and Crude Oils

The general results obtained in the last 20 years have been extensively reviewed very recently [2] and should be examined in detail because of the large amount of available data. The main aspects can be summarized as follows. First of all, the PO chain is significantly lipophilic, and only sightly hydrophilic, i.e., much less than a polyethylene chain. The PO unit is three times longer than a (CH_2_) methylene group in a surfactant typical *n*-alkyl tail; it thus considerably extends the surfactant penetration in oil and increases the interaction, particularly with polar oil segregated close to the interface. The first 2–4 PO groups stay close to the water phase, so the molecule is twisted in this zone, and is not really straight and perpendicular to the interface, as can be seen in Figure 5d [26,112]. In some cases, the accumulation of the PO groups outside the water can be considerable, in particular at the air surface where they are said to form rugby balls [40,113]. This arbitrary gathering of the first PO group in a short and twisted hydrated chain close to the water phase produces some disorder that helps avoid the surfactant’s rigidity and precipitation. A branched structure, such as the Guerbet double tail, was found to improve the extended surfactant interaction with oil phases, in particular di/triglycerides [14,114]. An increasing number of PO groups from five to 15 definitively increases the performance, which practically stays constant at higher PON. A PON term may be introduced in the HLD correlation but not as a linear term since it is also dealing with the surfactant hydrophobe part length (SAT) [2,27]. A more continuous change from hydrophilic to lipophilic parts, including an intermediate with an additional butylene oxide block between the alkyl tail and the propylene oxide block, improves, even more, the performance [28]. This is probably because it results in a very wide zone with a smooth variation of hydrophilicity, which is particularly appropriate to interact with crude oils containing many polar species containing functional groups bearing nitrogen, sulfur and oxygen atoms, such as asphaltenes, naphthenic acids, etc. [37,57,58,115,116].

The last benefit found in an extended surfactant is that being an intramolecular mixture between ionic and nonionic parts; a proper adjustment of the different blocks could present new opportunities such as insensitivity to temperature [117,118] or insensitivity to surfactant concentration [25] and robustness to electrolyte concentration and mixture variations, including new applications with complex biobased polar oils [29,38,119].

### 5.5. The Application of the HLD_N_ in Extended Surfactant Systems

After updating the hydrophilic–lipophilic deviation equation as the normalized HLD_N_ to obtain better accuracy, and after briefly presenting the extended surfactant structure and its advantages as performance is concerned, we will now present a few unconventional opportunities with extended surfactants.

**Figure 6:**
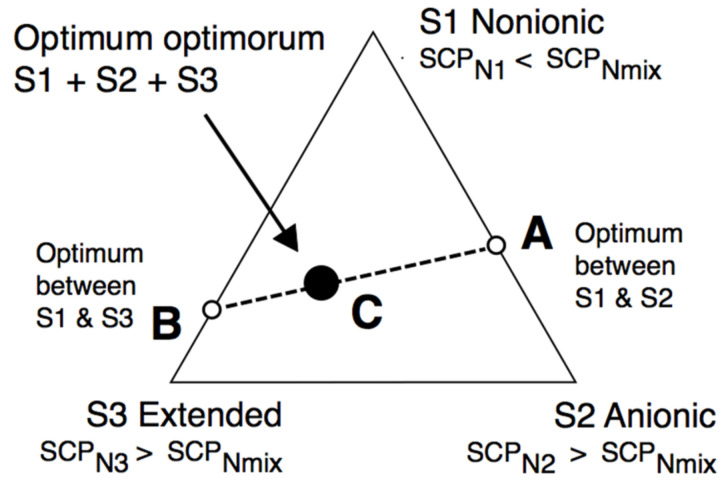
The synergetic optimum formulation is often attained with three surfactants when one of them is an extended species, at other constant variables (S, ACN, T, P, alcohol).

However, before that, we would like to show that with a more precise HLD_N_ equation and with a complex mixture of three surfactants, including an extended one, superior performance can be attained without needing a super expert researcher on the challenge. Figure 6 deals with a system in which the usual variables, i.e., the salinity, the oil nature, the temperature, the pressure and the absence of alcohol, are fixed. According to Equation (7) or (8), a certain value SCP_Nmix_ must be reached to obtain HLD_N_ = 0. It will be with a surfactant mixture, and it may be predicted by using three surfactants S1-S2-S3 in Equation (11), for instance, with SCP_N1_ < SCP_Nmix_ and SCP_N2_ as well as SCP_N3_ > SCP_Nmin_, so that an equation such as the following one can be found:

SCP_Nmix_ = X_1_ SCP_N1_ + X_2_ SCP_N2_ + (1−X_1_−X_2_) SCP_N3_ (11)

What we have reported as an easy-to-understand trick [119] is that the best way to mix three surfactants is to make two optimum formulations between two surfactants only, and then to join them. The first mixture is shown in Figure 6 between surfactant S1, which is more hydrophilic than the required mixture, and surfactant S2, which is more lipophilic, thus having an optimum formulation A with a mixture between S1 and S2, on the S1-S2 side. Moreover, the second mixture is between surfactant S1 (SCP_N1_ < SCP_Nmix_) and surfactant S3 (SCP_N3_ > SCP_Nmix_) with an optimum in point B on the S1−S3 side. A quick scan is then carried out along the AB line to find the *“optimum optimorum”* as would be said in Latin (black circle), which is the optimum between two previous optima.

HLD_Nmix_ = X_A_ HLD_NA_ + X_B_ HLD_NB_ = 0 + 0 = 0 (12)

In practice, it can be said from our numerous experiences that there is a second-order optimum at some point C, somewhere in the AB line or very close to it, which is better than the A and B cases because of a synergy effect. This is particularly systematic when one of the surfactants is an extended variety with a synergy effect with both simpler anionic and nonionic species. Moreover, it often presents a welcome insensibility to temperature, salinity, or surfactant concentration.

This complex but easy-to-understand practical technique to use synergy with extended surfactants shows the interest of knowledge and know-how in the formulation. We now present some unconventional cases.

## 6. Extended Surfactants for Drilling Fluids Applications

During the drilling of a well, various types of fluids are used, often called muds, because they are suspo-emulsions, i.e., water-in-oil or oil–water emulsions, stabilized by surfactants, and containing solid particles to control rheology and salts to control density [31,120,121]. Depending on their morphology, the muds are classified as oil-based muds (W/O) or water-based muds (O/W) [122,123,124].

Moreover, drilling fluids are mesophases that can be pre-manufactured or produced in situ and are used downhole for various treatments such as cleaning and removing non-polar materials in the reservoir production zones or eliminating well damage [125]. These fluids, also called spacers and/or pills, are used in oil and gas wells. These treatments are based on the solubilization of the non-polar oil material that exists in the well when oil-based mud is used as a drilling fluid [126,127,128].

### 6.1. Best Solubilization Drilling Fluid Cleaning through Single-Phase Wiping (at Optimum)

**Figure 7:**
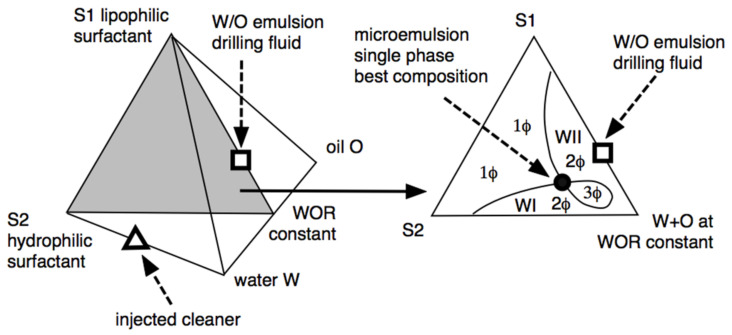
Ternary and quaternary diagrams showing the different phase behavior zones and the optimum formulation point (black circle) in which the single phase is attained at the minimum surfactant concentration [32].

We hereafter describe the particular case of the design of a spacer fluid between the drilling fluid and the cement. The most delicate problem is how the drilling fluid is an oil-based mud (W/O type emulsion) that is not miscible with the cement. In such a case, extended surfactant-containing brines have been designed that, in contact with the oil-based drilling mud, produce microemulsions or low interfacial tension systems [127]. This is possible because the composition of the drilling fluid is known, including the water phase brine. Figure 7 shows the different ways to obtain systems at or near HLD_N_ = 0 [31,32].

The phase diagram shows the lipophilic surfactant S1 used to stabilize the W/O emulsion drilling fluid at the top of the triangle in the ternary diagram, at a constant water/oil ratio that is roughly equal. The drilling fluid W/O emulsion (to be displaced) is indicated as a square on the S1-WOR = 1 side. The injected formulation (indicated in the left three-dimensional diagram as a triangle) is an aqueous solution of the hydrophilic surfactant S2, which is a special species [32,106] with a structure that helps the mixing between the in-situ drilling fluid (square) and the injected formulation (triangle), which takes place after several hours of contact in the center of the three-phase zone (3Φ) close to the black point in Figure 7. This is an optimal formulation, with a low tension in the 3Φ zone, not necessarily a single-phase microemulsion as indicated in the 1Φ zone [55,129]. An amazingly efficient injected cleaner was achieved by replacing conventional hydrophilic surfactants with a special extended S2 surfactant. The formation of a microemulsion occurs after several hours of contact between the brine containing S2 and the oil-based mud. The extended chain of S2 surfactants is characterized by the perfect gradation from the most hydrophilic to the most lipophilic portions, generating the exact balance. Therefore, it is possible to achieve high solubilization in mesophasic fluids (monophasic microemulsions) and find, in most cases, insensitivity to temperature, which makes it possible to use extended surfactants with a large variety of oils.

### 6.2. Extended Dendritic Surfactant for the Formulation of Oil-Based Muds and for Their Removal from the Well by Using a Fluid with a More Hydrophilic Extended Surfactant

**Figure 8:**
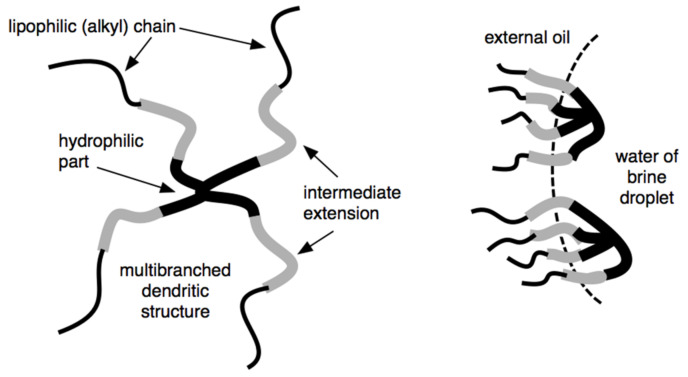
Dendritic extended surfactant (**left**) and its adsorption on a water drop interface in a drilling fluid W/O emulsion (**right**).

S1 surfactants with dendritic shape structure (Figure 8) for application as emulsifier or wetting agents for the design of oil-based muds were synthesized. By controlling the number of lipophilic chains attached to a hydrophilic head, it is possible to control the surfactant viscosity. It also introduces a spacer (intermediate polarity) between the hydrophilic head and lipophilic tail groups and makes it possible to increase the interaction with polar oils, which are the external phase of synthetic drilling fluids (Figure 8) [33].

Different types of extension (polyethoxylated, polypropoxylated, xylitol, polyglucoside groups, etc.) were studied in these dendritic extended surfactants. It was found that depending on this extended group, they can act preferentially as an emulsifier and/or a wetting agent of particles when added to an oil-based drilling fluid.

These modified surfactants can substantially reduce interfacial tension and thereby improve the interaction between the non-polar continuous phase and the brine droplet within the oil-based drilling fluid. By improving the interfacial properties, the oil-based drilling fluid can have greater water droplet size stabilization and higher lubricity, which increases in the penetration rate. In addition, a great advantage of the dendritic extended surfactant is that they can be used in smaller amounts than typical surfactants used in the oil-based drilling fluid. The modified surfactants can also produce emulsions of relatively low average droplet size (e.g., a mini-emulsion or nanoemulsion). The droplets may act as carriers for drilling fluid additives delivered downhole (Figure 8).

**Figure 9:**
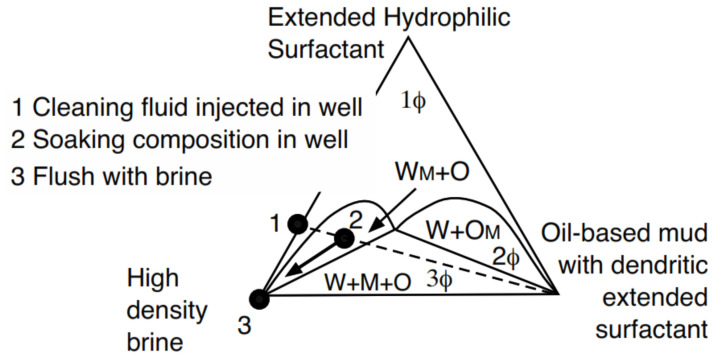
Cleaning process to remove the filter cake deposited during drilling.

Furthermore, it was easy to change the wettability of the wellbore wall, from oil-wet to water-wet, before the cementing operation by using a second fluid (2) designed with a more hydrophilic extended surfactant as seen in the W_M_+O two-phase zone (Figure 9), discussed in detail in the previously cited patents. In systems with high salinity and high temperature, the fluid indicated as (1) is first pumped. Then, the well is closed for 24 h and the fluid becomes the formulation indicated as (2). The high-density brine fluid (3) is then injected and the filter cake is dissolved, producing a clean space to inject cement.

## 7. Improved Performance and Robustness in Crude Oil Dewatering and Dehydration

In petroleum production, crude oil dewatering is one of the key stages previous to transportation and refining [130,131,132]. Water in crude oil emulsions, which happens in most cases, is known to be stabilized mostly by asphaltenes. Asphaltenes are large molecules (700 to 1000 Da) with a polyaromatic core, branching, and functional groups (heteroatoms O, N, S) that make them the more polar species in crude oil, and therefore, those with interfacial activity [133,134,135,136]. Consequently, they adsorb at the W/O interface, with different aggregation states, forming a film with high mechanical resistance that hinders the coalescence of water in crude oil emulsions at HLD_N_ > 0 [137].

Emulsion breaking and destabilization, and therefore crude oil dehydration, is known to occur with a minimum stability exactly at HLD_N_ = 0 [70,132,138]. Thus, physicochemical formulation can be changed according to the variables that can be used (for example, the type of surfactant used, temperature and cosurfactant) [34,130,132]

Nevertheless, it is not exactly known when the minimum stability is lowest at optimum formulation because the emulsion properties—such as the viscosity of the external phase, as well as viscoelasticity, droplet size, and in the case that there are particles, their wettability—influence the destabilization and emulsion lifetime [62,139]. Several phenomena are known to influence emulsion stability at a certain distance from the optimal formulation—for example, the effect of aromatic solvents which hinder asphaltenes interfacial activity. Other variables are temperature that would generate a lower viscosity and faster sedimentation. Hence, it is not known if they act as well as HLD_N_ = 0, which would be the preferred method to assess at a fast destabilization of emulsions. It is now known that in addition to the very low interfacial tension and the equal partitioning of the surfactant species between oil and water, two phenomena occur at HLD_N_ = 0 that influence emulsion stability: (i) the minimum of interfacial elasticity [36,72] and (ii) the concomitant effective negligible Gibbs–Marangoni effects (due to the accelerated exchanges of surfactant between the bulk and the interface) [36,73]. Nevertheless, demulsifier formulations are much more complex than the model systems that have been studied, which was discussed in a very recent report [140].

Therefore, an efficient dehydration formulation must incorporate surfactants that not only adsorb rapidly at the interface but that can also change physicochemical formulation from HLD_N_ > 0 to HLD_N_ = 0 and form a WIII system [34,132,138]. In order to generate such a change, dehydration formulations are usually composed of a surfactant with an SCP_N_ < 0, a hydrocarbon solvent (usually aromatic or naphthenic), a cosurfactant alcohol that adsorbs rapidly at the interface (e.g., sec-butanol) [141], accelerating the surfactant exchanges between the bulk and the interface [142,143], and also some kind of cosolvent that can act as a linker between the surfactant and the oil [35,144,145,146].

### 7.1. The Application of Extended Surfactants for Efficient Breaking of Crude Oil in Water Emulsions

Extended surfactants have been shown to present a molecular structure that mimics this complex mixture, specifically tailored to interact with large polar oils as the asphaltene fraction that is segregated near the interphase [13,23,35]. Thus, they facilitate the emulsion breaking at HLD_N_ = 0 and increase performance, resulting in robustness at optimum formulation [146,147,148]. This was recently verified when comparing a very common nonionic surfactant (NPEO6) and an extended surfactant molecule as C_12_(PO)_14_(EO)_2_SO_4_Na [36]. Extended surfactants of the ionic (propoxylated and ethoxylated sulfate, carbonate, or phosphate sodium salts) and of the nonionic type have been proven to generate fast emulsion breaking in water in heavy crude oil emulsions [35,146]. This occurrence is mainly due to the effects produced by the first three to four PO groups near the interphase, which have been shown to be partially hydrated, generating some twisting of the molecule, resulting in some disorder that favors the interfacial film breaking.

Additionally, there is the effect of the longer part of the PO chain, which is branched and not necessarily linear, but considerably extends the tail to penetrate in the asphaltene segregated layer near the interface [103] and in the oil bulk and results in avoiding precipitation. It could be said that the adsorbed extended surfactant produces a much longer interaction zone in the oil phase. Thus, in practice, more interaction between the surfactant and the oil side molecules weakens the intermolecular attraction per tail unit length, thus avoiding the formation of liquid crystal or solid films such as precipitated asphaltenes clusters [2,146].

As with other surfactants used in demulsifier formulations, extended species can be more or less hydrophilic by changing the structure. In the early 1990s, it was proposed [132] that the destabilization of the crude oil emulsion is maxima at HLD_N_ = 0, i.e., when the asphaltenic surfactant positive SCP_NA_ is compensated by the demulsifier compensation effect SCP_ND,_ making the proper mixture appear at the interface. The hydrophilic effect of the demulsifier depends on the SCP_ND_ as well as the fraction interfacially adsorbed, i.e., X_D_. As explained elsewhere, here are two variables with a result roughly depending on the product X_D_ SCP_ND_ [130,131]. This means that more surfactant is required for a less hydrophilic one. Therefore, a very hydrophilic extended surfactant will be more effective than a less hydrophilic one, although if it is too hydrophilic, it can go to the water more than to the interface. Moreover, low stability is an important feature, but the robustness, i.e., a wide instability zone, is also important since the asphaltenes demulsifiers adsorbed at the interface cannot be easily measured.

**Figure 10:**
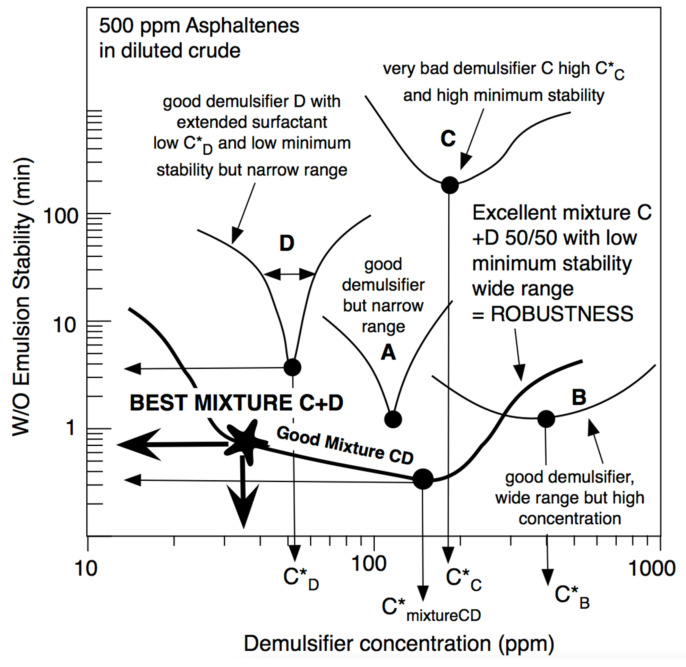
Performance (as minimum concentration to attain the minimum stability) and robustness (as the range of low stability zone) of the water in diluted crude emulsion when using different surfactant (A, B, C, D) and a proper C + D mixture, including extended surfactants.

Figure 10 shows different situations with different demulsifiers in an emulsion of water in diluted crude oil with 500 ppm of asphaltenes. This figure indicates three performances. The first one is the C*_D_ concentration of the demulsifier to attain the minimum stability. It is generally lower if the surfactant is more hydrophilic, i.e., if its SCP_N_ is more negative. In practice and in model dilutions of crude oil, a proper value should be lower than C*_D_~100 ppm. The second performance is the value of minimum stability, which is indicated in Figure 10 as the time to attain a 60% separation of the water phase, in this case with no electrical effect. In practice and with the shown experiments, a maximum of two to three minutes is desirable. Lastly, a third performance is often neglected, even if it is quite important. It was called the robustness [35], and it corresponds to some estimation of the possible range in which the stability is minimum. For instance, in case A of Figure 10, C*_D_ is about 100 ppm and the minimum stability is about a minute, and it thus seems to be a good case. However, the minimum stability takes place in a very narrow concentration range, and a very small inaccuracy in the applied concentration can change the selection. Extended surfactant demulsifier D is even better since the required concentration is quite low, but the robustness is poor.

In case B, very low stability is attained and a rather wide zone is found, i.e., good robustness is delivered, but in this case, the required concentration is too high, probably because the surfactant is too hydrophilic.

Some systematic studies [146] indicated that formulations with extended surfactants with SCP_N_ << 0 (far from optimum, i.e., very hydrophilic, and very effective to change HLD_N_) showed a low performance and low robustness. The trend seems to be that SCP_N_ < 0 extended surfactants (SCP_N_ negative but not too much) need slightly more demulsifier but with a higher performance (lower stability) and better robustness.

A special feature was presented recently by Delgado et al. [146], where the mixture of a high-performant extended surfactant (D) mixed with a poorly performant conventional surfactant (C) can generate remarkable robustness that allows reaching very low emulsion stability at very low demulsifier addition (Figure 10). It is shown to happen because the C + D (50/50) mixture has a very wide and low stability zone. Even if the lowest stability is found at 150 ppm, it is seen that there is still a low stability, 30 to 40 ppm, even if it is slightly higher than the minimum. This occurrence is of utmost importance at the industrial setup, at the surface of the oil well when demulsifier dosage cannot be very precise [36].

It is not known for sure if this synergy is happening systematically, but several examples in our industrial work have shown that extended surfactants are efficient in mixing effect with conventional surfactants to help to break emulsions at optimum formulation. A particular work performed by our laboratory with a petroleum company indicated that an extended surfactant with no anionic head but with a purely polyethoxylated (long) one was particularly efficient at a very low concentration, i.e., 10 ppm [140].

### 7.2. Extended Surfactants Robust Systems to Decrease Interfacial Elasticity at Low Demulsifier Concentration

The effect of higher performance and robustness of extended surfactants as demulsifiers was corroborated with a high-end innovative equipment—i.e., the oscillating spinning drop interfacial rheometer [73,149]. Extended surfactants of the C_12_(PO)_14_(EO)_2_SO_4_Na type were used in emulsified water in heavy crude oil (diluted in cyclohexane) systems [36,72]. The extended surfactants were shown to decrease the dilational elasticity (~10^−2^) and to break emulsion very quickly (<1 min) with low surfactant dosage (200 ppm), and the occurrence of a large robustness zone with low stability of the emulsions as in Figure 10 for the C + D mixture. Actually, this performant formulation is studied to better understand the mechanism of water in crude oil emulsions destabilization and relate HLD_N_ = 0 with lowering the asphaltene film’s rigidity [132,138]. It has been recently suspected, although without scientific publications, that the phase angle at HLD_N_ = 0 can be very low with the presence of extended surfactants in the interfacial film [36] due to the formation of a microemulsion lens around the droplet in the rotating capillary [140,150].

## 8. Future Perspective

The main feature of extended surfactants is that they exhibit a linker-like intramolecular mixture with the central polypropylene oxide extension. As discussed in the first studies, this results in higher solubilization of large (polar) oil molecules within the microemulsion domains, including crude oils and biobased oils. This ionic/nonionic structure allows their use at high salinities (with no precipitation) and with a relative insensibility to high temperatures. Nevertheless, the most significant advance is to exhibit a long surfactant molecule without precipitation or liquid crystal formation.

New environmental regulations are being pushed forward everywhere in the world, with a clear trend toward biobased alternatives to crude oils, hydrocarbons, or synthetic solvents, in particular with high-water-content microemulsions. New extended surfactant-like molecules, including biodegradable functional sugar head groups that started to be developed at the FIRP Laboratory in the 2000s and more biocompatible extensions, seem to be good candidates for future alternatives.

Biobased complex oil mixtures (from wood, algae, or other plant-based sources) and the use of biobased surfactant mixtures would require the use of more generalized formulation expressions that could be applied in academic and industrial settings. The HLD_N_-related tools, which are currently approximately used to formulate products for enhanced oil recovery, pharmaceuticals, detergent, cosmetics, oil extraction from seeds, and even water remediation, have to be improved not only with a single scale as with HLD_N_ and the SCP_N_. More variables have to be included, e.g., with surfactant having more than two parts (head and tail) and with widely mixed natural oils (with something more significant than EACN) and real brines with different electrolytes. Of course, it is probable that 20 to 30 variables will be too many to have useful expressions at hand, even with a computer, and that a proper combination of theoretical models and empirical determinations will appear in the best formulation in the years to come.

## Figures and Tables

**Table 2:** Different cases of equations indicating the changes of two formulation variables to deviate from optimum formulation and to return to it (ΔHLD = 0) with coefficients found in experimental data.

HLD Equation–Surfactant Type
ΔHLD_1_ = ΔLnS − 0.16 ΔACN = 0 for alkylbenzene sulfonates ΔHLD_2_ = ΔLnS − 0.19 ΔACN = 0 for alkyltrimethyl ammonium chlorides ΔHLD_3_ = ΔLnS − 0.07 ΔACN = 0 for alkyl hexapropyleneoxide diethylenoxide sulfates ΔHLD_4_ = 0.33 ΔSAT − ΔEON = 0 for ethoxylated *n*-alcohol with EON~5 and T~25 °C ΔHLD_5_ = 0.13 ΔS − ΔEON = 0 for ethoxylated *n*-alcohol with EON~5 and T~25 °C ΔHLD_6_ = 2.25 ΔSAT − ΔACN = 0 for ethoxylated *n*-alcohol with EON~5 ΔHLD_7_ = − 0.24 ΔACN − ΔEON = 0 for ethoxylated *n*-alcohol with EON~5 ΔHLD_8_ = − ΔT − 20 ΔACN = 0 for *n*-alkyl sulfates ΔHLD_9_ = − ΔT − 14.3 ΔACN = 0 for alkylbenzene sulfonates ΔHLD_10_ = ΔT − 4 ΔACN = 0 for ethoxylated nonionic (EON~5–6 & T~20–30 °C) ΔHLD_11_ = ΔT − 1.4 ΔACN = 0 for ethoxylated nonionic (EON~8–9 & T~70 °C) ΔHLD_12_ = ΔT − 0.90 ΔACN = 0 for ethoxylated nonionic (EON~10–11 & T~80–90 °C) ΔHLD_13_ = − ΔGN − 0.12 ΔACN = 0 for polyglyceryl monolaurate (GN~5–6) ΔHLD_14_ = ΔLnS − 0.14 ΔPON = 0 for alkyl polypropyleneoxide diethylenoxide sulfates

where S is the salinity in wt% NaCl, EON is the exact or average number of ethylene oxide groups, SAT is the surfactant *n*-alkyl tail length in carbon atom number, T is the temperature in °C, and GN is the number of glyceryl group in polyglyceryl monolaurate oligomers.

**Table 3:** Various cases of equations indicating the changes of two formulation variables to deviate from optimum formulation and return to it (ΔHLD = 0) with the same −1 coefficient in front of the ΔACN so that all the equations have the same scale.

HLD_N_ Equation−Surfactant Type
ΔHLD_N1_ = 6.25 ΔLnS − ΔACN = 0 for alkylbenzene sulfonates ΔHLD_N2_ = 5.26 ΔLnS − ΔACN = 0 for alkyltrimethyl ammonium chlorides ΔHLD_N3_ = 14.3 ΔLnS − ΔACN = 0 for alkyl hexapropyleneoxide diethylenoxide sulf. ΔHLD_N4_ = 1.4 ΔSAT − 4.2 ΔEON = 0 for ethoxylated *n*-alcohol (EON~5 & T~25 °C) ΔHLD_N5_ = 0.55 ΔS − 4.2 ΔEON = 0 for ethoxylated *n*-alcohol (EON~5 & T~25 °C) ΔHLD_N6_ = 2.25 ΔSAT − ΔACN = 0 for ethoxylated *n*-alcohol with EON~5 ΔHLD_N7_ = − 4.2 ΔEON − ΔACN = 0 for ethoxylated *n*-alcohol with EON~5 ΔHLD_N8_ = − 0.05 ΔT − ΔACN = 0 for *n*-alkyl sulfates ΔHLD_N9_ = − 0.07 ΔT − ΔACN = 0 for alkylbenzene sulfonates ΔHLD_N10_ = 0.25 ΔT − ΔACN = 0 for ethoxylated nonionic (EON~5–6 & T~20–30 °C) ΔHLD_N11_ = 0.70 ΔT − ΔACN = 0 for ethoxylated nonionic (EON~8–9 & T~70 °C) ΔHLD_N12_ = 1.1 ΔT − ΔACN = 0 for ethoxylated nonionic (EON~11 & T~80–90 °C) ΔHLD_N13_ = − 8.3 ΔGN − ΔACN = 0 for polyglyceryl monolaurate (GN~5–6) ΔHLD_N14_ = 1.2 ΔPON − ΔACN = 0 for alkyl polypropyleneoxide PON sulfates

## Data Availability

Not available.
